# Targeting Oxidative Stress With Auranofin or Prima-1^Met^ to Circumvent p53 or Bax/Bak Deficiency in Myeloma Cells

**DOI:** 10.3389/fonc.2019.00128

**Published:** 2019-03-06

**Authors:** Benoit Tessoulin, Geraldine Descamps, Christelle Dousset, Martine Amiot, Catherine Pellat-Deceunynck

**Affiliations:** ^1^CRCINA, INSERM, CNRS, Université d'Angers, Université de Nantes, Nantes, France; ^2^L'Héma-NexT, i-Site NexT, Université de Nantes, Nantes, France; ^3^SIRIC ILIAD, Angers, Nantes, France; ^4^Service d'Hématologie Clinique, Unité d'Investigation Clinique, CHU de Nantes, Nantes, France

**Keywords:** Prima-1^Met^, APR-246, auranofin, ROS, venetoclax

## Abstract

Prima-1^Met^ (APR-246) was previously shown to be dependent on glutathione inhibition and on ROS induction in cancer cells with mutated or deleted *TP53*. Because this ROS induction was, at least in part, due to a direct interference with the thioredoxin reductase enzyme, we investigated whether activity of Prima-1^Met^ could be mimicked by auranofin, an inhibitor of the thioredoxin reductase. We thus compared the activity of auranofin and Prima-1^Met^ in 18 myeloma cell lines and in 10 samples from patients with multiple myeloma or plasma cell leukemia. We showed that, similar to Prima-1^Met^, the activity of auranofin was not dependent on either *TP53* status or p53 expression; was inhibited by N-acetyl-L-cysteine, a ROS scavenger; displayed a dramatic synergy with L-buthionine sulfoximine, an irreversible inhibitor of glutathione synthesis; and induced cell death that was not dependent on Bax/Bak expression. These data showed that auranofin and Prima-1^Met^ similarly overcome cell death resistance in myeloma cells due to either p53 deficiency or to mitochondrial dysfunction.

## Introduction

Mutations and/or deletions of *TP53* are associated with resistance to treatments in multiple myeloma as in most B-cell malignancies (1). *TP53* mutation and deletion are associated in B-cell malignancies suggesting that bi-allelic alterations of *TP53* are involved in resistance, although overall survival of patients with lymphoma or myeloma appears more significantly related to mutations than to deletion (2, 3). Mutations of *TP53* induced a more rapid development of spontaneous tumors than the deletion of *TP53* (4). Moreover, some mutations are characterized by a gain of function, making mutant forms of the p53 protein interesting therapeutic targets (5). Given the importance of folding for p53 activity and the existence of temperature-dependent mutations, chemical molecules able to change the conformation of p53 and to restore its transcriptional activity were screened (6). During the last 15 years, several molecules were isolated for their efficacy to induce cell death in *TP53* mutated cells and some of these molecules were shown to interact with the mutant p53 protein (7, 8). However, the p53 dependency of several p53 reactivating molecules, such as RITA and Prima-1^Met^, is debated as both drugs killed cancer cells independently from *TP53* status and p53 expression (9, 10). Indeed, it was recently demonstrated using CRISPR/Cas9 technology that the cell response to RITA, which is a DNA damaging drug, depended on FANCD2 expression (11). On the other hand, Prima-1^Met^ has been shown to decrease glutathione and to induce ROS independently from p53 expression or mutations (10, 12, 13), at least by directly interfering with thioredoxin reductase, a central enzyme of the detoxifying redox pathway (14). These results prompted us to further investigate whether auranofin, an inhibitor of the thioredoxin reductase, displayed a Prima-1^Met^-like activity. We therefore investigated activity and death mechanism of auranofin in myeloma cell lines and primary myeloma cells characterized for *TP53* status. We showed that activity of auranofin and Prima-1^Met^ correlated in myeloma cells and that both drugs induced a Bax/Bak-independent cell death.

## Materials and Methods

### Human Myeloma Cell Lines (HMCLs) and Primary Samples

Eighteen HMCLs used for this study, i.e., 7 HMCLs with a wild-type *TP53* status (MDN, NCI-H929, NAN9, NAN11, XG3, XG6, XG7), 8 HMCLs with a missense *TP53* mutation (JIM3, KMS12PE, LP1, NAN10, OPM2, U266, XG2, XG5) and 3 HMCLs with a *TP53* indel leading to the lack of mRNA and/or protein expression (JJN3, L363, NAN7). All HMCLs have been extensively characterized (10, 15, 16). *TP53* status was performed by direct sequencing of RT-PCR products (16) and by whole exon sequencing (17). After obtaining informed consent, blood or bone marrow samples from patients with MM were collected at the Department of Hematology of the Nantes University Hospital (MYRACLE cohort, ethical approval NCT03807128, Benaniba et al., submitted). Plasma cells were obtained after gradient density centrifugation and FISH was performed as previously described (9, 18).

### Reagents and Antibodies

Prima-1^Met^ was purchased from Santa Cruz Biotechnology (CliniSciences, Nanterre, France), L-buthionine sulfoximine (BSO), auranofin and N-acetyl-L-cysteine were purchased from Sigma-Aldrich (Saint-Quentin Fallavier, France). Anti-CD138-PE monoclonal antibody was purchased from Beckman Coulter (Villepinte, France), Annexin V-FITC was purchased from ImmunoTools (Friesoythe, Germany), anti-Bak, anti-Bax and anti-actin were purchased from BD-Biosciences (Le Pont de Claix, France), Cell Signaling (Ozyme, Montigny-le-Bretonneux, France) and Millipore (Guyancourt, France), respectively.

### siRNA Experiments

Transient *BAX* or *BAK* silencing was performed in LP1 myeloma cells (100 pmol siRNA/3 × 10^6^ cells) using lipofectamine RNAiMax (Thermo Fischer Scientific, Saint-Herblain, France), as previously reported (19).

### Cell Death Assay

The cell lines or mononuclear cells from patients' samples (500,000 cells/ml) were incubated with Prima-1^Met^ or auranofin with different concentrations as indicated within the legends of the figures. Cell death was assessed by Annexin V staining in cell lines and by the loss of CD138 staining in primary myeloma cells (10, 19–21). The fluorescence acquisition and analysis were performed using a FACsCalibur with Cell Quest (Becton Dickinson) or FlowJo (Ashland, OR, USA) software, Cytocell core facility (SFR Bonamy, Nantes, France).

### Statistical Analyses

The statistical analyses were performed using GraphPad Prism 7.

## Results

### Sensitivity of Myeloma Cell Lines to Prima-1^Met^ and Auranofin Correlated

**Figure 1:**
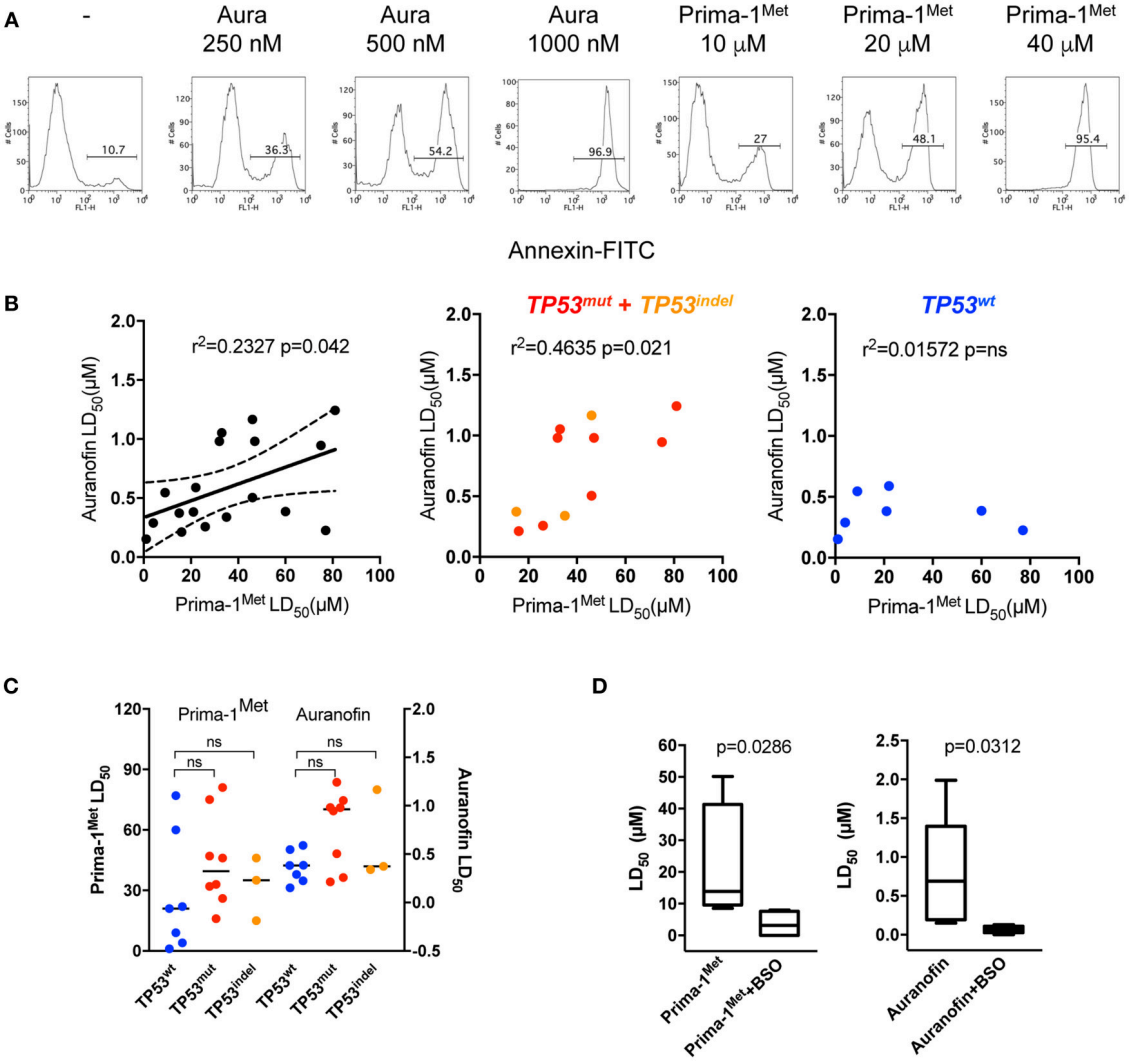
HMCLs were similarly sensitive to Prima-1^Met^ and to auranofin. **(A)** OMP2 cells (500 000/ml) were incubated with or without (-) increasing concentrations of auranofin or Prima-1^Met^ for 2 days as indicated in the figure, and cell death was assessed by Annexin V staining. **(B)** The LD_50_ (lethal dose 50) values of auranofin were plotted against the LD_50_ values of PRIMA-1^Met^ in 18 HMCLs (left). The graphs of the middle and right panels represent the correlations between the two drugs in *TP53*^*Abn*^ and *TP53*^*wt*^ HMCLs, respectively. The statistical analyses were performed using the Pearson test. **(C)** The LD_50_ values of auranofin and PRIMA-1^Met^ were analyzed according to *TP53*^wt^, *TP53*^mut^, and *TP53*^indel^ statuses. The statistical analyses were performed using the Mann-Whitney test. **(D)** HMCLs were incubated with increasing concentrations of each drug with or without BSO (500 μM) for 2 days. Cell death was assessed by Annexin V staining. The statistical analyses were performed using the Wilcoxon matched-pairs signed-rank test.

We assessed the efficacy of auranofin, an inhibitor of thioredoxin reductase, in comparison with Prima-1^Met^ in 18 HMCLs. We determined the lethal dose 50 (LD_50_) of auranofin and Prima-1^Met^ to HMCLs using Annexin V staining at day 2, as illustrated in Figure 1A. Figure 1B (left panel) shows a positive correlation between LD_50_ values for auranofin and Prima-1^Met^ (*p* = 0.042, *r*^2^ = 0.2327, Pearson test); however, auranofin displayed higher activity (about 80-fold) compared to Prima-1^Met^ (median values were 0.4 and 32.5 μM, respectively). Notably, the correlation was essentially supported by HMCLs displaying abnormal *TP53* status (Figure 1B, middle and right panels, Pearson test), although activity of auranofin and Prima-1^Met^ was not dependent on p53 mutations or expression (Figure 1C). Using the CellMinerCDB portal (https://discover.nci.nih.gov/cellminercdb/), which provides pharmacologic sensitivity of cancer cell lines, we confirmed that activity of another inhibitor of the thioredoxin reductase (PX-12) also correlated with activity of Prima-1^Met^ in myeloma cell lines (Figure S1).

Because it was previously shown that Prima-1^Met^ synergized with BSO, an irreversible inhibitor of GSH synthesis, we determined whether auranofin also synergized with BSO (10, 12). Six HMCLs (JJN3, MDN, OPM2, XG5, XG6, and U266) were cultured with increasing concentrations of Prima-1^Met^ or auranofin with or without BSO (500 μM) and LD_50_ values were determined. Both Prima-1^Met^ and auranofin strongly synergized with BSO, and LD_50_ values were decreased by ~ 4- and 10-fold, respectively (Figure 1D).

### Auranofin and Prima-1^Met^ Induced ROS-Dependent Bax/Bak-Independent Cell Death

**Figure 2:**
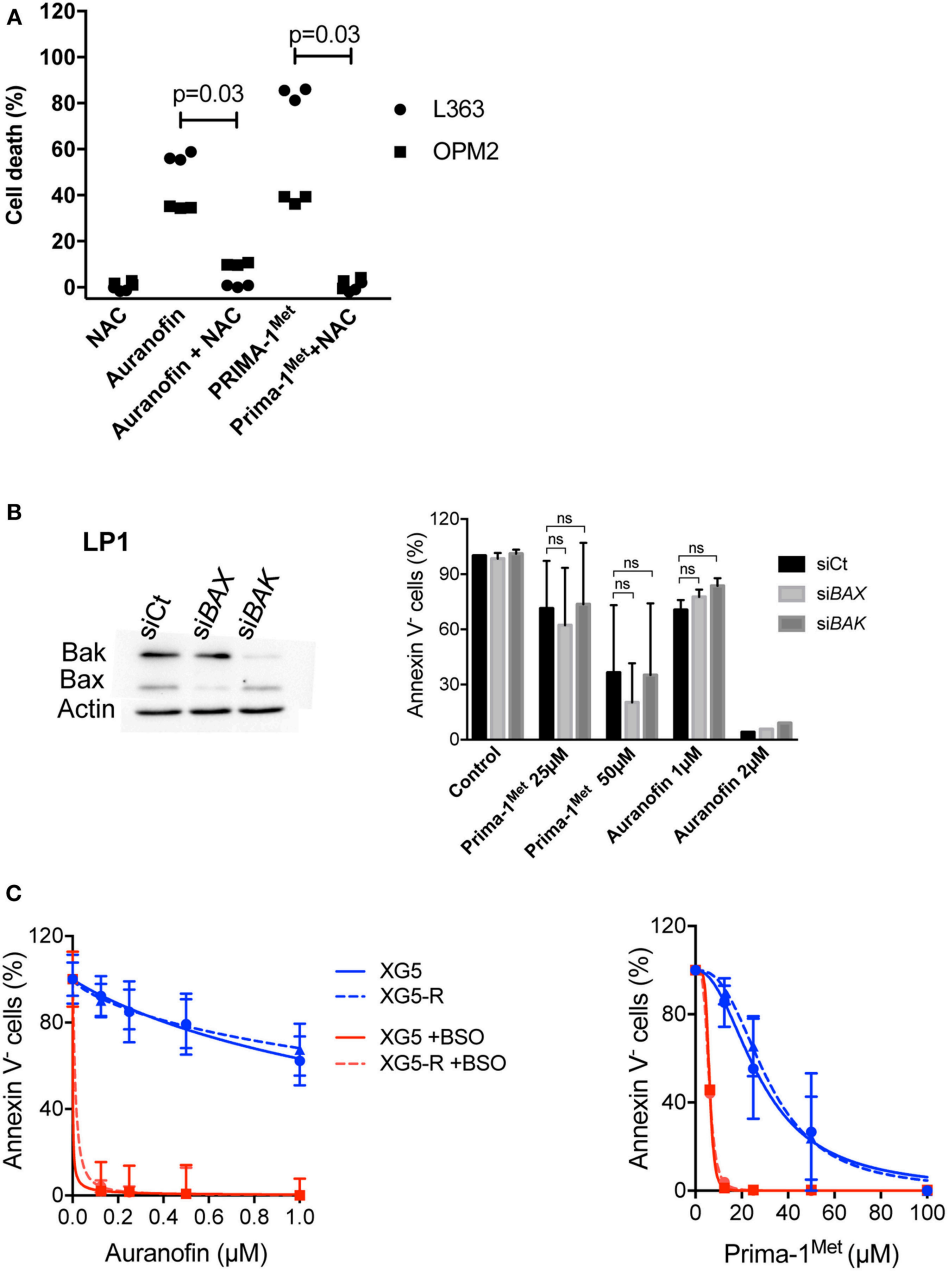
Auranofin and Prima-1^Met^ induced ROS-dependent BAX/BAK-independent cell death. **(A)** L363 and OPM2 cell lines (500 000 cells/ml) were incubated for 2 days with auranofin (2000 and 1000 nM, respectively) or Prima-1^Met^ (80 and 40 μM, respectively) with or without NAC (0.5 mM). Cell death was assessed by Annexin V staining. The data represent 3 independent experiments. The statistical analyses were performed using the Wilcoxon matched-pairs signed-rank test. **(B)** LP1 cells were transfected with siRNA against *BAX or BAK1* for 2 days and sensitivity to each drug was assessed. The statistical analyses were performed using the two-way ANOVA test with multiple comparisons. **(C)** Auranofin and Prima-1^Met^ LD_50_ values were determined in XG5 parental cells and in venetoclax resistant XG5-199R cells. Cells were incubated with increasing concentrations of auranofin or Prima-1^Met^ with or without 500 μM BSO for 2 days, and cell death was assessed by Annexin V staining.

We previously demonstrated that Prima-1^Met^ induced ROS-dependent cell death in myeloma cells that was prevented by N-acetyl-L-cysteine (NAC) (10). Because auranofin has been shown to induce ROS production, we thus assessed whether NAC was also able to inhibit cell death induced by auranofin (22). As shown in Figure 2A in L363 and OPM2, the addition of NAC inhibited cell death induced by auranofin and Prima-1^Met^ by 86% (*p* = 0.03) and 95% (*p* = 0.03), respectively. Prima-1^Met^ was shown to induce apoptosis that was independent from Bax/Bak: Prima-1^Met^ induced lipid peroxidation that mediated mitochondrial permeabilization, cytochrome C release and activation of caspases (13). To determine the role of Bax/Bak in auranofin-induced cell death, we performed *BAX*/*BAK1* silencing in LP1 cells. A decrease in Bax or Bak expression did not significantly modify cell responses to either drug (Figure 2B). Moreover, the venetoclax-resistant XG5-199R HMCL, in which the expression of both apoptosis executors was lost, remained as sensitive as the parental XG5 cell line to both drugs (Figure 2C) (23). These results showed that auranofin and Prima-1^Met^ induced ROS-dependent, Bax/Bak-independent cell death.

### Sensitivity of Primary Myeloma Cells to Prima-1^Met^ and Auranofin Correlated

**Figure 3:**
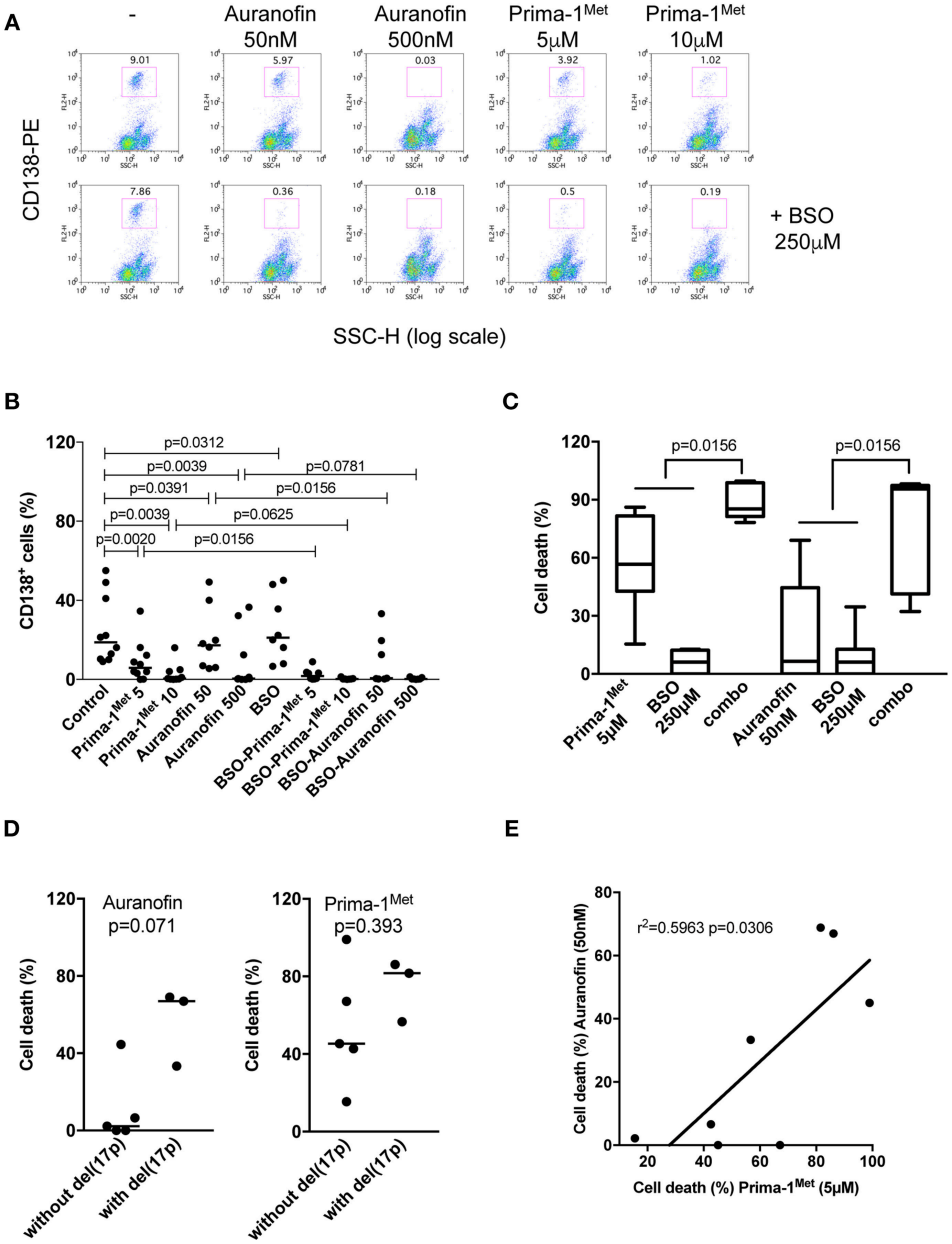
Primary myeloma cells were similarly sensitive to Prima-1^Met^ and to auranofin. **(A)** Mononuclear cells from patient #4 with myeloma were incubated for 24 hours with or without (-) Prima-1^Met^ (5 or 10 μM) or auranofin (5 or 10 μM) and stained with anti-CD138-PE. Viability was assessed by CD138 expression. **(B)** Mononuclear cells from 10 samples were incubated for 24 hours with Prima-1^Met^ (5 or 10 μM) or auranofin (5 or 10 μM) with or without BSO (250 μM), and stained with anti-CD138-PE. The statistical analyses were performed using the Wilcoxon matched-pairs signed-rank test. **(C)** Cell death induced by Prima-1^Met^ (5 μM, samples #3,4,5,6,8,9, and 10), auranofin (50nM, samples #3,4,5,6,7,9, and 10) or BSO (250 μM, samples #3-10) was compared to cell death induced by combinations (combo) of Prima-1^Met^ or auranofin with BSO. The statistical analyses were performed using the Wilcoxon matched-pairs signed-rank test. **(D)** Cell death induced by auranofin (50 nM) or Prima-1^Met^ (5 μM) was analyzed according to del(17p) status. The statistical analyses were performed using the Mann-Whitney test. **(E)** Myeloma cell death induced by 5 μM of Prima-1^Met^ was plotted against cell death induced by 50 nM auranofin. Correlation was performed using the Pearson test.

We assessed the activity of each drug with or without BSO in 10 samples from patients with either multiple myeloma (MM) or plasma cell leukemia (PCL) with or without the deletion of the short arm of chromosome 17, del(17p). Mononuclear cells were incubated with different concentrations of Prima-1^Met^ or auranofin with or without BSO (250 μM). Cell death was determined by the loss of CD138 expression, Figure 3A. Indeed, cell death could not be monitored by Annexin V staining as the loss of myeloma viability was associated with the loss of expression of the plasma cell specific CD138 expression, as illustrated in Figure S2 (10, 19, 20). Both drugs significantly induced myeloma cell death (Figure 3B; Table 1). The median values of cell death induced by Prima-1^Met^ (5 or 10 μM) were 68% (*p* = 0.0020) and 97% (*p* = 0.0039), respectively, and the median values of cell death induced by auranofin (50 or 500 nM) were 20% (*p* = 0.0391) and 97% (*p* = 0.0039), respectively. Although BSO (250 μM) induced a weak decrease in cell viability (median cell death 7.5%, *p* = 0.0312, Figure 3B), it significantly synergized with both Prima-1^Met^ (5 μM) and auranofin (50 nM). The cell death median values of the combination of BSO with Prima-1^Met^ or with auranofin vs. the sum of cell death induced by Prima-1^Met^ or auranofin plus BSO were 85.2% vs. 62.8% (*n* = 7, *p* = 0.0156, 1.24-fold increase) and 95.6% vs. 12.7% (*n* = 7, *p* = 0.0156, 6.41-fold increase), respectively (Figure 3C). The sensitivity of samples to both drugs was not different in samples with or without the deletion of the short arm of chromosome 17, Figure 3D. Auranofin (50 nM) and Prima-1^Met^ (5 μM) displayed correlated activity in myeloma samples (*n* = 8, *r*^2^ = 0.5963, *p* = 0.0306), Figure 3E.

**Table 1:** Prima-1^Met^ and Auranofin induced cell death in primary myeloma cells.

**Sample**			**CD138**^****+****^ **cells (%)**									
**N^**°**^**	**Status**	**del(17p)**	**Control**	**Prima-1^**Met**^ 5 μM**	**Prima-1^**Met**^ 10 μM**	**Auranofin 50 nM**	**Auranofin 500 nM**	**BSO 250 μM**	**Prima-1^**Met**^ 5 μM + BSO**	**Prima-1^**Met**^ 10 μM + BSO**	**Auranofin 50 nM + BSO**	**Auranofin 500 nM + BSO**
1	MM	nd	12.9	0.1	0.1	nd	0.4	nd	nd	nd	nd	nd
2	MM	–	10.3	3.1	0.3	nd	0.1	nd	nd	nd	nd	nd
3	PCL	+	22.3	4.1	nd	6.9	nd	22.4	3.3	nd	0.6	nd
4	MM	+	9.0	3.9	1.0	6.0	0.0	7.9	0.5	0.2	0.4	0.2
5	MM	–	16.1	8.8	4.0	16.4	0.3	16.1	3.0	0.3	0.3	0.1
6	MM	–	21.3	12.2	5.0	19.9	12.5	20.0	3.6	0.1	12.5	0.1
7	MM	–	10.1	0.1	0.1	5.6	1.1	6.6	0.1	0.1	0.3	0.2
8	PCL	–	55.0	7.6	0.2	18.2	0.4	50.1	0.2	0.2	nd	0.2
9	PCL	+	40.9	34.6	16.1	40.0	32.3	35.7	8.9	1.4	19.6	1.0
10	MM	–	49.0	16.1	0.4	49.2	36.6	48.0	0.6	0.4	33.2	1.0

*Peripheral blood or bone marrow samples were obtained from patients with plasma cell leukemia (PCL) or Multiple Myeloma (MM)*.

*Deletion of the short arm of chromosome 17, del(17p), was performed by FISH*.

*Mononuclear cells were incubated overnight with Prima-1^Met^, Auranofin with or without BSO, as indicated within the table*.

*nd, not done*.

## Conclusion

In this paper, we showed that the sensitivity of myeloma cells to auranofin correlated with sensitivity to Prima-1^Met^ in both HMCLs and in primary myeloma cells from patients with MM or PCL, and that auranofin was more efficient than Prima-1^Met^. Although p53 competent HMCLs were very sensitive to both drugs, the activity of Prima-1^Met^ or auranofin was not dependent on *TP53* status, highlighting that targeting the ROS/GSH pathway was efficient in cells expressing mutant p53 protein or lacking p53 expression. Prima-1^Met^ and auranofin induced Bak/Bax-independent cell death and were efficient in myeloma cells resistant to the Bcl2-specific BH3-mimetic venetoclax. These data showed that auranofin, as Prima-1^Met^, overcomes resistance to cell death mediated by either p53 deficiency or by mitochondrial loss of priming in myeloma cells. Both drugs appear thus of particular interest for resistance *in vivo*. Prima-1^Met^ (APR-246) is under clinical evaluation in ovarian cancers with mutated *TP53* or in refractory (*TP53*-mutated) myeloid neoplasms, alone or in combination. Auranofin, which was used to treat patients with arthritis, was recently shown to be able to eliminate side populations and to enhance ibrutinib efficacy in solid cancer cells (22, 24). These recent findings are in favor of assessing auranofin in myeloma patients resistant to current therapies as well as resistant to the Bcl2-specific BH3 mimetic venetoclax.

## Data Availability

All datasets generated for this study are included in the manuscript and/or the supplementary files.

## Author Contributions

BT designed the study, performed experiments, and participated in the writing of the article. GD performed experiments. CD and MA provided XG5 venetoclax-resistant cells. CP-D designed the study and wrote the article. All authors approved the manuscript.

### Conflict of Interest Statement

The authors declare that the research was conducted in the absence of any commercial or financial relationships that could be construed as a potential conflict of interest.
