# Supplementary figures and images for: Targeting Oxidative Stress With Auranofin or Prima-1Met to Circumvent p53 or Bax/Bak Deficiency in Myeloma Cells
Source: Front Oncol. 2019 Mar 6;9:128. doi: 10.3389/fonc.2019.00128 (PMC6414792; doi:10.3389/fonc.2019.00128)

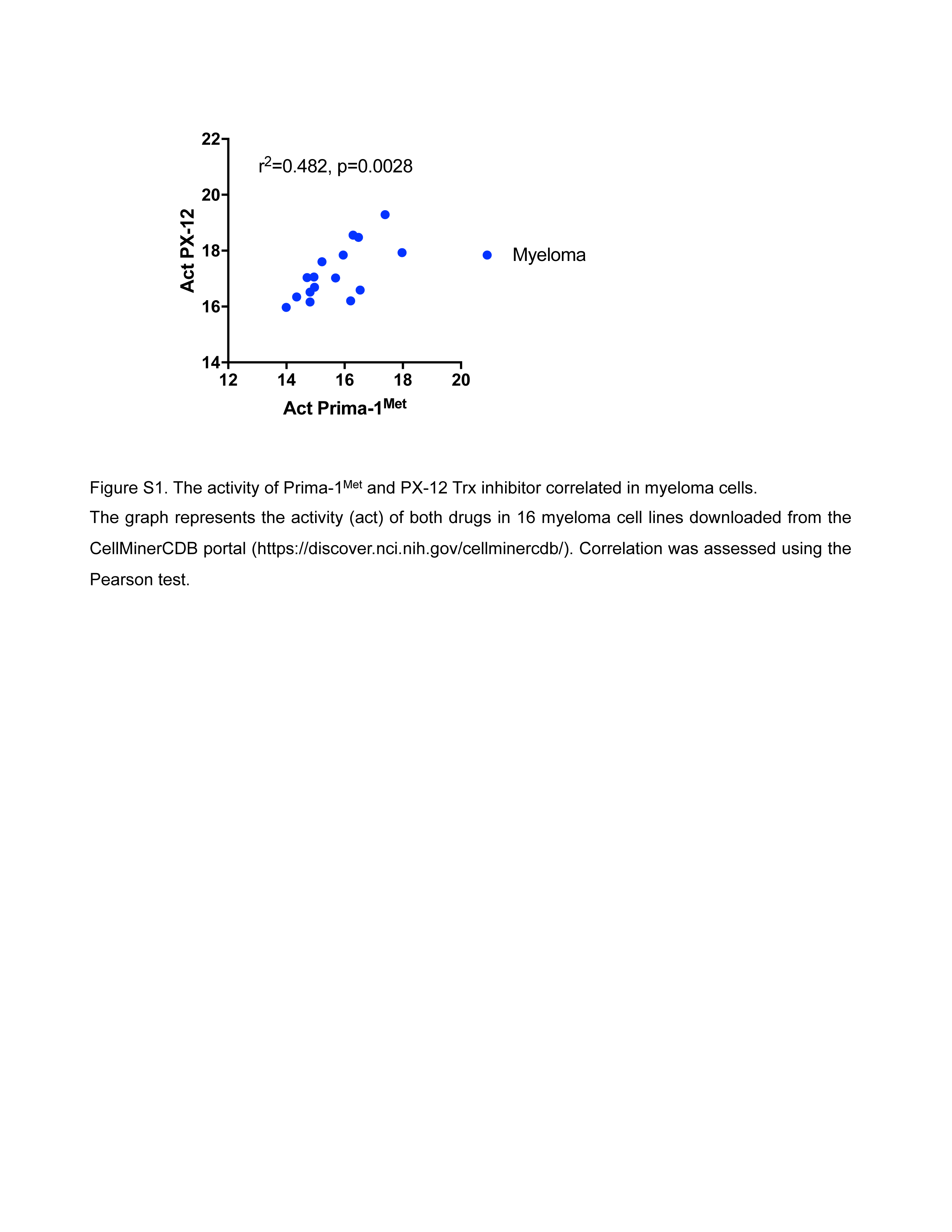

Supplement: Supplementary file 1 [file Image_1.TIF]

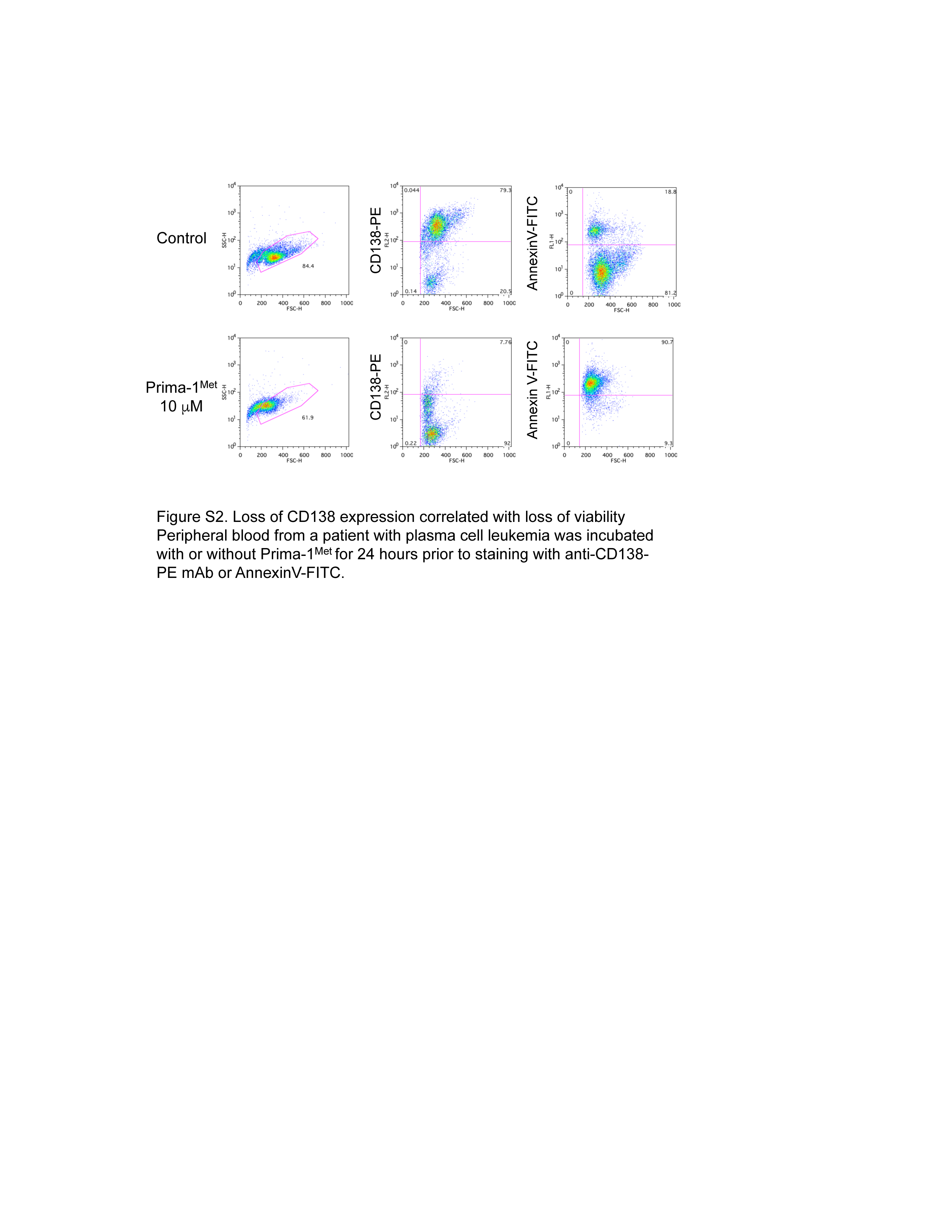

Supplement: Supplementary file 2 [file Image_2.TIF]
